# FINE TUNING OF A PRETRAINED GAIT MODEL FOR FRAILTY CLASSIFICATION PREDICTION

**DOI:** 10.1093/geroni/igae098.3984

**Published:** 2024-12-31

**Authors:** Laura McDaniel, Ime Essien, Yuxiang Guo, Ayush Gupta, Vineet Shenoy, Peter Abadir, Rama Chellappa

**Affiliations:** Johns Hopkins University, Baltimore, Maryland, United States; Johns Hopkins University, Baltimore, Maryland, United States; Johns Hopkins University, Baltimore, Maryland, United States; Johns Hopkins University, Baltimore, Maryland, United States; Johns Hopkins University, Baltimore, Maryland, United States; Johns Hopkins University, Baltimore, Maryland, United States; Johns Hopkins University, Baltimore, Maryland, United States

## Abstract

This study presents a multimodal gait model designed to identify the frailty status of individuals based on their gait observed in video footage. Frailty is defined as a clinically recognizable state of increased vulnerability resulting from age-related declines in reserve and function across multiple interrelated physiological systems. These risks manifest as an increased likelihood of falls, hospitalization, need for long-term care, disability, and mortality. Although deep learning has advanced in medical applications, its potential in early frailty detection through gait analysis is limited by the need for extensive data, which is currently limited. To address this issue, we leveraged the OpenGait framework, GaitBase, a pretrained gait model typically used for gait surveillance and recognition applications, by fine-tuning it with medical data for frailty classification. Data were collected using the marker-less Qualisys Motion Capture system, resulting in a dataset of 64 individuals classified by frailty status—non-frail, frail, and pre-frail—according to the Fried Frailty Phenotype. Detectron2 models were used for panoptic image segmentation and detection, generating silhouette and box predictions from video frames. By integrating both silhouette and RGB data, our model enhances robustness and generalization across the three frailty classes. To address the challenges of a small dataset, we employed data augmentation, hyperparameter tuning, and cross-validation to improve classification accuracy. This model aims to advance frailty classification using deep learning, offering a time-efficient method for clinicians to assess frailty. Future steps include incorporating metadata such as height and weight to further enhance prediction accuracy.

